# The Chromatin “Landscape” of a Murine Adult β-Globin Gene Is Unaffected by Deletion of Either the Gene Promoter or a Downstream Enhancer

**DOI:** 10.1371/journal.pone.0092947

**Published:** 2014-05-09

**Authors:** Brenda Cadiz-Rivera, George Fromm, Christina de Vries, Jennifer Fields, Kathleen E. McGrath, Steven Fiering, Michael Bulger

**Affiliations:** 1 Department of Pediatrics, University of Rochester Medical Center and Center for Pediatric Biomedical Research, Rochester, New York, United States of America; 2 National Institute for Environmental Health Sciences, NIH, Research Triangle Park, North Carolina, United States of America; 3 Department of Microbiology and Immunology, Dartmouth Medical School, Hanover, New Hampshire, United States of America; Schulze Center for Novel Therapeutics, Mayo Clinic, United States of America

## Abstract

In mammals, the complex tissue- and developmental-specific expression of genes within the β-globin cluster is known to be subject to control by the gene promoters, by a locus control region (LCR) located upstream of the cluster, and by sequence elements located across the intergenic regions. Despite extensive investigation, however, the complement of sequences that is required for normal regulation of chromatin structure and gene expression within the cluster is not fully defined. To further elucidate regulation of the adult β-globin genes, we investigate the effects of two deletions engineered within the endogenous murine β-globin locus. First, we find that deletion of the β2-globin gene promoter, while eliminating β2-globin gene expression, results in no additional effects on chromatin structure or gene expression within the cluster. Notably, our observations are not consistent with competition among the β-globin genes for LCR activity. Second, we characterize a novel enhancer located 3′ of the β2-globin gene, but find that deletion of this sequence has no effect whatsoever on gene expression or chromatin structure. This observation highlights the difficulty in assigning function to enhancer sequences identified by the chromatin “landscape” or even by functional assays.

## Introduction

The transcriptional regulation of protein-coding genes often results from the combined regulatory inputs of multiple *cis*-acting DNA sequences, which together comprise the transcriptional control region. For most genes in prokaryotes and unicellular eukaryotes, the transcriptional control region is limited entirely to sequences located in the region just upstream of the site of transcription initiation. In contrast, many metazoan genes, if not the majority of them, are influenced by a more numerous and more widely dispersed set of DNA sequences. Notably, metazoan genes are often regulated by distal sequences, such as enhancers, which are capable of influencing transcriptional events at the promoter and during transcriptional elongation despite being located large distances (tens to hundreds of kilobases) away [1], [2]. This property makes the complete identification and characterization of the transcriptional control regions of metazoan genes a difficult task. The locations of enhancers can be predicted via several features of chromatin structure – DNase I hypersensitive sites, specific histone modification “signatures” like histone H3 K4 monomethylation, noncoding sequence conservation, etc. – and functional enhancer sequences can be characterized via gain-of-function assays like the transient reporter gene or colony assays, but the assignment of a distal enhancer to a given gene depends, in the end, upon genetic assays that manipulate endogenous gene loci.

**Figure 1:**
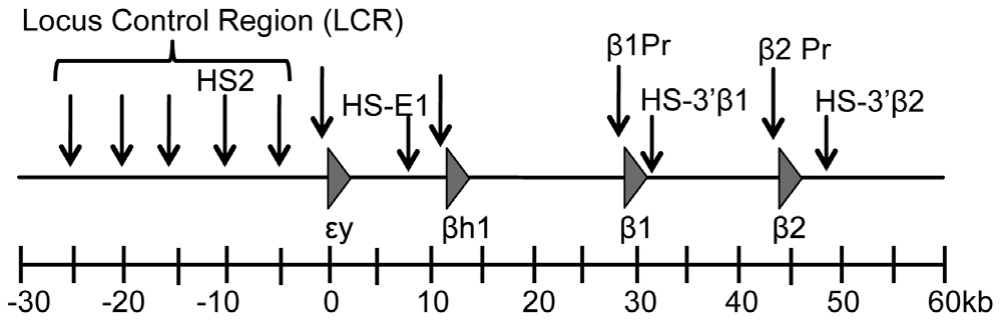
The murine β-globin locus. The murine β-globin locus is depicted with the β-globin genes represented by triangles and known or putative regulatory sequences, to which DNaseI HSs have been mapped, by vertical arrows. The scale at the bottom is drawn with the transcription start site for the ey-globin gene set as +1 bp.

In this vein, the mammalian β-globin locus probably represents the single most intensely investigated gene locus in the transcription field. The β-globin genes reside in a single cluster in most mammals and are expressed in an erythroid-specific fashion. Expression from within the cluster also changes during mammalian development: erythropoiesis initiates with a transient wave of primitive erythrocytes that originate in the embryonic yolk sac, after which the primary site of erythropoiesis moves successively to the fetal liver and adult bone marrow, where definitive erythrocytes differentiate and enucleate prior to their release into the circulation [3], [4]. The murine β-globin locus is comprised of four genes – the εy- and βh1-globin genes, which are expressed specifically in primitive erythrocytes in the early embryo and silenced in definitive erythroid cells, and the β1- and β2-globin genes, which are expressed at low levels during primitive, and very high levels during definitive erythropoiesis (Figure 1). This developmental specificity is primarily encoded in the β-globin promoter-proximal regions, although additional sequences distributed within the cluster appear to contribute as well [5], [6], [7]. Beyond this, however, high-level expression of these genes in erythroid cells at all developmental stages requires a set of distal regulatory elements spread over a region of 25–30 kb that is located 5′ of the gene cluster and termed the locus control region (LCR) [8], [9].

Thus, transcriptional control of the β-globin genes is accomplished in part by the promoter regions of the genes themselves, by additional intergenic sequences and by the presence of the LCR. In particular, in the absence of the LCR, transcription is either completely eliminated (in human) [10] or drastically reduced (in mouse) [8], and the best evidence from mouse lines harboring the LCR deletion is that the primary effect is on transcriptional elongation [11]. Other features of the active β-globin locus in erythroid cells, however, are not affected by loss of the LCR. These include the pattern of histone modifications within the gene cluster: active β-globin gene promoters are associated with histone hyperacetylation and H3K4 methylation, neither of which is affected upon deletion of the LCR [8], [12], [13]. Moreover, the β-globin genes reside within larger domains characterized by hyperacetylated histones and H3K4 dimethylation [12], [14], [15], [16]. We have termed these regions “hyperacetylated domains,” and recently we demonstrated that a novel enhancer located between the two embryonic-specific murine β-globin genes, HS-E1, is required for normal formation of the domain that encompasses these genes in primitive erythrocytes. In addition, deletion of the element from the endogenous murine β-globin locus results in 60-80% decreases in embryonic β-globin expression levels [6].

The discovery and initial characterization of HS-E1, coupled with additional observations of potential regulatory sequences located outside of the gene promoters or the LCR, demonstrates that the transcription control region for the β-globin gene cluster, as complex as it already might appear to be, remains undefined. Notably, two distinct hyperacetylated domains occur over the β1- and β2-globin genes, and are present in both primitive and definitive erythroid cells and qualitatively identical in both despite the dramatic difference in expression levels of these genes between the two stages. Deletion of previously identified control sequences, including the LCR and HS-E1, has no effect on chromatin structure in the vicinity of the adult β1- or β2-globin genes [6], [12], [14]. The identity of *cis*-acting DNA sequences involved in the establishment and maintenance of these domains remains obscure. To this end, we characterize the effects of deletion of two *cis*-acting DNA regulatory elements within the hyperacetylated domain that encompasses the murine β2-globin gene. First, we analyze the effect of deleting the β2-globin gene promoter region on domain formation and expression of other members of the β-globin gene cluster. Second, we identify and characterize a novel enhancer located 3′ of the β2-globin gene, termed HS-3′β2, and analyze the effect of its deletion from the endogenous locus as well.

## Results

**Figure 2:**
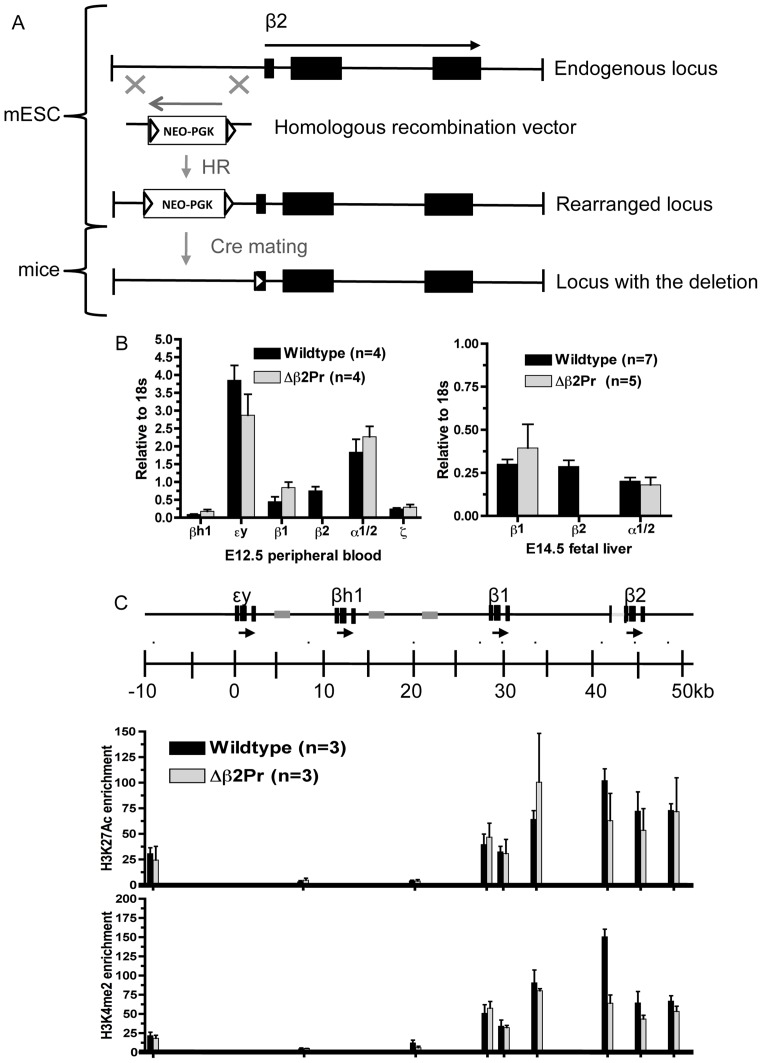
Deletion of the β2-globin gene promoter. A. Schematic showing the strategy for deletion of the promoter region of the β2-globin gene. Exons are shown as black boxes, while the *neo* resistance marker cassette, with expression driven by the PGK promoter, is shown as a white box. Triangles indicate *loxP* sites. B. Analysis of gene expression. Bar graphs show expression of the indicated globin genes relative to 18S rRNA. Values derived from wildtype mice are shown by black bars, and from homozygous mutant mice by gray bars. The number of embryos used for each set of measurements is indicated as n. Error bars show standard error of the mean. C. Analysis of histone modifications. A schematic of part of the β-globin locus is shown at the top, with genes indicated by horizontal arrows and their exons by black boxes. The gray boxes indicate the positions of β-globin pseudogenes within the locus. The scale is drawn with the transcription start site for the ey-globin gene set as +1 bp. The small black dots above the scale indicate the positions of PCR amplimers used for ChIP analysis. Brackets in the schematic of the β-globin locus at the top show the location of the deleted region. The bar graphs show fold enrichments for occupancy by histones exhibiting the indicated covalent modifications, relative to the average of 2 negative control probes (NPTXR, OLFR78) derived from inactive gene loci. In addition, discrepancies between individual ChIP experiments were eliminated by normalizing to the average of control probes within active gene loci (Glycophorin A, ferrochelatase, Cl-/HCO3- anion exchanger glycoprotein, Rh-assocaited glycoprotein). Black bars denote values derived from wildtype mice, and gray bars values from homozygous mutant mice, with the number of separate embryos analyzed indicated as n. Asterisks denote data points for which student t-test p values are <0.05.

The β-globin gene promoters are obviously crucial to the proper expression of the genes themselves, but their contributions to chromatin structure elsewhere in the locus have rarely been evaluated. To evaluate the contribution of the adult β-globin gene promoters to chromatin modifications associated with this region, we engineered the deletion of the β2-globin gene promoter by homologous recombination in embryonic stem (ES) cells (Figure 2A). The deletion removed ∼600 bp of DNA, up to the first 25 bp of the coding region in exon 1, and replaced this sequence with a neomycin resistance marker (*neo*) flanked by binding sites (*lox*) for Cre recombinase. Mice were derived from these ES cells and the *neo* marker removed by mating with a Cre-expressing mouse line.

We analyzed the expression of β-globin genes by isolating RNA from primary erythroid cells derived from homozygous mutant and wildtype littermates obtained from matings of heterozygous parents. As expected, deletion of the β2-globin gene promoter eliminates expression of the gene (Figure 2B). In addition, however, we carefully measured the expression of the other β-globin genes, both in primitive (E12.5 peripheral blood) and definitive (E14.5 fetal liver) erythroid cells. The dominant model for LCR-mediated activation of the β-globin genes posits that the LCR interacts directly with the gene promoters, and that in turn it can only interact with one promoter at a time. Thus, the β-globin gene promoters are thought to compete for the activity of the LCR, and numerous transgenic studies have given support to this model [17], [18], [19]. Studies analyzing the endogenous locus, while not as numerous, have not been as supportive of this model – deletion of the εy-globin gene promoter failed to result in increased expression of βh1, and vice versa [20], [21]. We find that the β1- and β2-globin genes similarly do not appear to compete for LCR activity. Deletion of the β2-globin promoter does not result in a significant increase in expression of β1-globin, either in primitive or definitive erythroid cells. Notably, this holds despite the fact that in E14.5 fetal liver, fully 50% of β-globin expression is normally comprised of β2.

To evaluate the role of the β2-globin gene promoter in regulation of the hyperacetylated domain that encompasses the gene, we performed chromatin immunoprecipitation (ChIP) using antibodies that recognize histone H3 acetylated at lysine 27 (H3K27Ac) or dimethylated at lysine 4 (H3K4Me2). We have previously shown that these modifications are specifically enriched throughout the hyperacetylated domains within the β-globin locus [12], [14], [22]. ChIP was performed on dissociated E14.5 fetal livers obtained from homozygous mutant and wildtype littermates. Nonspecific rabbit IgG and a control histone H3 (C-terminus) antibody did not result in significant enrichments over background at any probe used. We find that a probe located immediately upstream of the deleted region appears to show a significant decrease in each of these modifications (p<0.05), but at no other location within the locus does the pattern of enrichment differ between mutant and wildtype mice (Figure 2C). Thus, the β2-globin gene promoter, as with the promoters for the embryonic εy- and βh1-globin genes, is not required for the proper establishment or maintenance of the histone modification patterns that characterize the murine β-globin locus *in vivo*.

**Figure 3:**
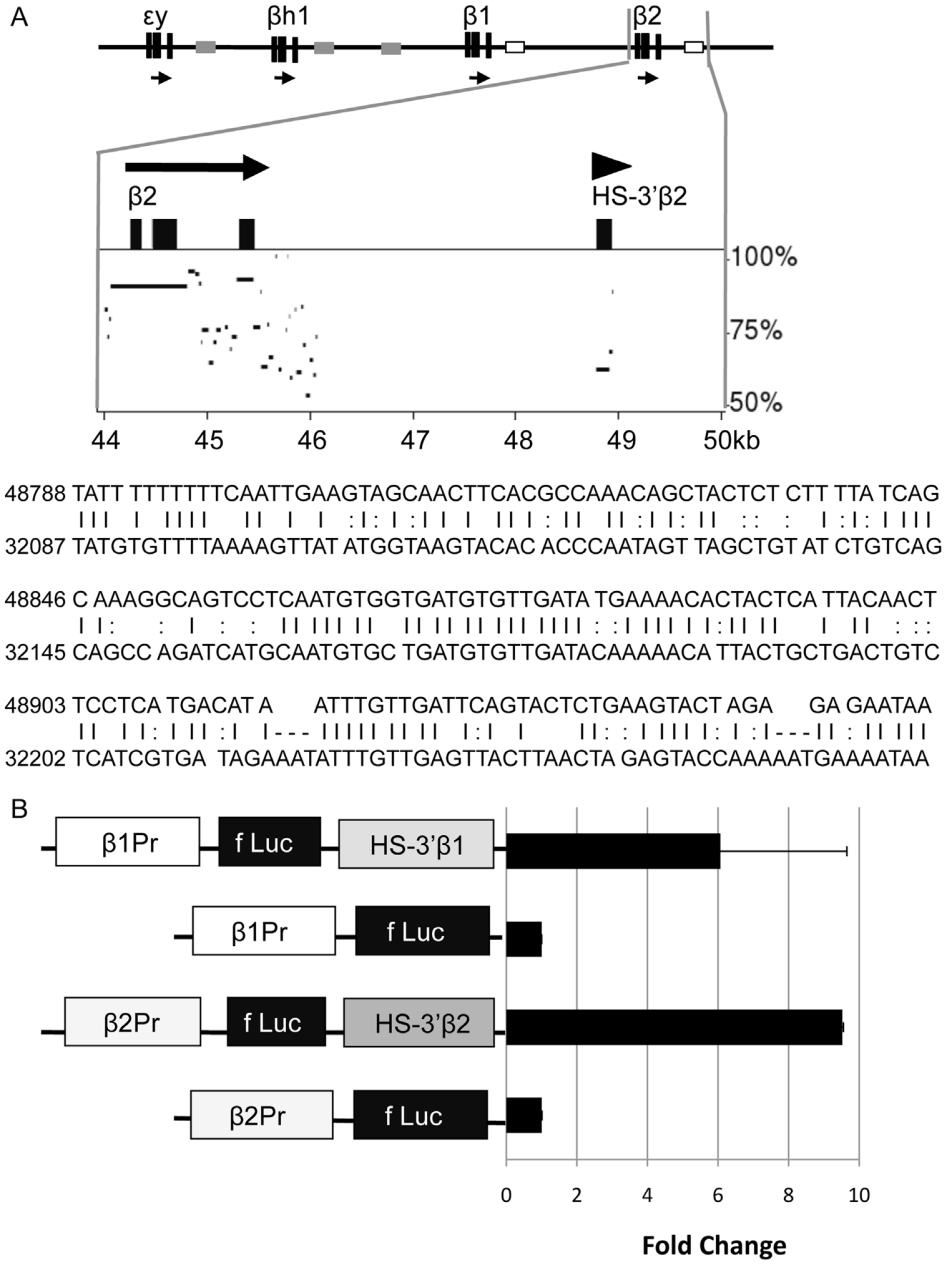
Identification of novel enhancers downstream of the adult murine β-globin genes. A. Sequence homology between HS-3′β1 and HS-3′β2. The murine β-globin locus is shown at the top, with the region near the β2-globin gene expanded beneath it. The locations of the 3′β1 and 3′β2 enhancers are indicated by open boxes. The graph represents a similarity plot for the region indicated as compared to the region near the β1-globin gene, generated by Pipmaker [27]. Beneath this, the sequences of both regions are shown to illustrate the homology. B. Transient transfection analysis of HS-3′β1 and HS-3′β2. The basic arrangement of sequences – gene promoter (derived from the β1- or β2-globin genes), firefly luciferase cDNA, and candidate enhancer – is shown on the left. The bar graph shows the results of luciferase assays performed on extracts of MEL cells transfected with the indicated constructs after 48 hrs. To normalize, the fluorescence measured for the enhancerless constructs was set as 1.0.

Based on the example of the embryonic εy- and βh1-globin genes, and the similar failure of the adult β2-globin gene hyperacetylated domain to require the presence of the gene promoter, we hypothesize that domain formation over the β2-globin gene results from the activity of a distinct sequence, presumably an enhancer, located within the domain or at its boundaries. We had previously mapped a DNaseI hypersensitive site (HS) downstream of the β2-globin gene, which we now term HS-3′β2 [14]. DNaseI HSs generally result from small (100–200 bp) regions within which transcription factors bind and nucleosomes are excluded [23], [24]. Notably, at the approximate location to which we mapped HS-3′β2, we observe a small region of sequence homology with a similar region located downstream of the β1-globin gene (Figure 3A). A DNaseI HS, which we term HS-3′β1, maps to this latter sequence as well [14]. These sequence and structural features identify HS-3′β1 and HS-3′β2 as candidate enhancers.

To establish the function of HS-3′β1 and HS-3′β2 as distal enhancers, we placed each sequence immediately downstream of a luciferase reporter gene driven by either the β1- or β2-globin gene promoters and evaluated their activity in transient transfection assays (Figure 3B). We find that both regions confer significant and reproducible increases in reporter gene expression upon transfection in murine erythroleukemia (MEL) cells. Thus, HS-3′β1 and HS-3′β2 represent novel transcriptional enhancers as defined by their activity in a canonical gain-of-function assay.

**Figure 4:**
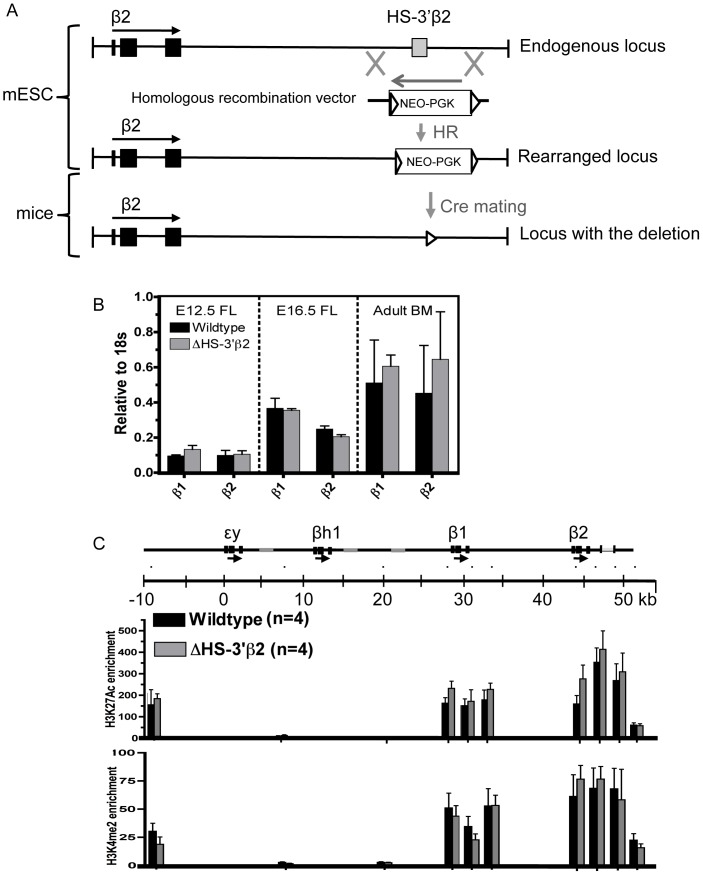
Deletion of HS-3′β2. A. Schematic showing the strategy for deletion of HS-3′β2, as in Fig. 2A. The region defined as HS-3′β2 – by HS mapping, sequence homology with HS-3′β1 and activity in the transient transfection assay – is indicated by the gray box. B. Analysis of gene expression. Bar graphs show expression of the indicated genes at the indicated timepoints as in Fig. 2B. FL = fetal liver, BM = bone marrow. C. Analysis of histone modifications. Bar graphs show fold enrichments for the indicated histone modifications at the indicated positions within the β-globin locus as in Fig. 2C.

To investigate the activity of HS-3′β2 in its native context, we engineered the deletion of this region from the endogenous murine β-globin locus by homologous recombination in ES cells. As with the β2-globin promoter deletion, the deleted sequence (amounting to 316 bp roughly centered over the region homologous to HS-3′β1) was replaced with a *neo* marker flanked by *lox* sites (Figure 4A), and mice were derived from an ES cell subclone harboring the replacement. The *neo* marker was removed by mating with a Cre-expressing mouse line.

To assess the effects of the deletion on β-globin gene expression, we purified RNA from definitive erythroid cells derived from homozygous wildtype or mutant mice (fetal liver from various timepoints and adult mouse bone marrow) and measured the expression of the adult β-globin genes by quantitative RT-PCR using primers that specifically amplify regions within the β1- or β2-globin transcripts. Surprisingly, we find that deletion of HS-3′β2 has no significant effect on expression of either adult β-globin gene in any of the erythroid cells we examine (Figure 4B). This remains the case when we examine expression in primitive erythroid cells derived from embryonic yolk sac. In addition, we performed flow sorting of definitive erythroid cells from E16.5 fetal liver to isolate cells at distinct stages of erythroid maturation, in order to determine if HS-3′β2 is required during a specific window of the differentiation process, but we observed no changes in gene expression at any stage examined (data not shown).

To evaluate the potential contribution of HS-3′β2 to hyperacetylated domain formation over the region, we performed ChIP analysis of histone modifications within the β-globin locus in E14.5 fetal liver in homozygous wildtype and mutant littermates (Figure 4C). Consistent with the lack of effect on β-globin mRNA levels, we do not observe any significant change in levels of H3K27Ac or H3K4Me2 anywhere in the β-globin locus upon deletion of HS-3′β2.

## Discussion

The complement of *cis*-acting regulatory sequences required for proper expression of the β-globin genes has not been fully defined. Previous studies of the murine β-globin locus established the fundamental requirement for the LCR in achieving high-level expression in erythroid cells, but even in the absence of this region active chromatin marks are still established at the genes, the hyperacetylated domains that normally encompass them still form, RNA polymerase II is still recruited to the promoters and a small amount of expression (1-4% of normal) still takes place [11], [12]. In an attempt to delineate the contributions of other known and potential regulatory sequences to these phenomena, we analyzed deletions within the endogenous locus of the β2-globin gene promoter and of a putative enhancer located downstream of the β2-globin gene.

Deletion of the β2-globin gene promoter unsurprisingly results in complete elimination of β2-globin gene expression. We fail to observe a significant effect on expression of the remaining β1-globin gene, however. In fetal liver, our own measurements indicate that β2-globin comprises ∼50% of transcription within the locus. The complete loss of this substantial fraction of normal β-globin mRNA levels is not replaced by a compensatory increase in β1-globin transcription. Models of LCR function that invoke competition for its activity would predict that β1-globin expression should increase dramatically in this context, since the competing β2-globin promoter has been removed. Our results suggest either that such models are inaccurate, or that competition for LCR activity involves β2-globin-proximal sequences other than the promoter region.

In addition, deletion of the β2-globin promoter has no other measurable effect on chromatin structure within the locus, with the limited exception of a small region located immediately upstream of the deletion. This result is consistent with our previous analyses of deletions of the promoters of the embryonic εy- and βh1-globin gene promoters [20], [21], and suggests that β-globin promoters in general do not exert any appreciable nonlocal effects on chromatin structure.

We are particularly interested in the formation of the hyperacetylated domain that encompasses the β2-globin gene in erythroid cells. Our prior experience with the domain encompassing the embryonic εy- and βh1-globin genes indicated that neither the LCR nor the gene promoters were required for its formation [12], and in fact we were able to identify a novel, primitive erythroid-specific enhancer, termed HS-E1, located between the two genes. Deletion of HS-E1 resulted in the loss of histone hyperacetylation throughout the εy-/βh1-globin region [6]. With this model in mind, we attempted to define potential enhancer regions within the β2-globin domain that might similarly serve to function in domain formation. HS-3′β2 represents the most obvious candidate for such a function – we have mapped a DNaseI HS to this location [14], and importantly the sequence exhibits enhancer activity in a transient reporter gene assay (Figure 3B). By the canonical definition of an enhancer, HS-3′β2 is one. It was therefore surprising to find no measurable effect of deletion of this element from within the murine β-globin locus. Regardless of its function in artificial assays, or of characteristic structural features that we can map to this sequence *in vivo*, it does not appear to be required for normal gene expression or for chromatin structure. This could imply that HS-3′β2 simply does not act as an enhancer within the β-globin locus, despite its activity in the reporter gene assay. It could also imply that the regulatory activity provided by HS-3′β2 is redundant with the activity of another element within the β2-globin domain. We are currently pursuing additional gain-of-function assays in order to evaluate all of the sequences within the β2-globin domain for enhancer function. Unfortunately, due to the presence of the domain itself, scans of the region for enhancer-specific histone modifications are minimally informative, since such modifications – including histone H3 lysine 4 monomethylation (H3K4Me1) and H3K27Ac – are significantly elevated throughout the domain, precluding the delineation of distinct peaks.

In this respect, our results highlight a significant problem with the identification of distal enhancers, namely their assignment to specific target genes. Recent high-throughput approaches to enhancer discovery, primarily utilizing putative enhancer-specific histone modifications and DNaseI sensitivity, have suggested that the mammalian genome harbors an enormous number of such elements, and furthermore that they represent the primary sequence determinants of cell type-specific gene expression [1]. For the purposes of analysis, most such studies make a “nearest neighbor” assumption that any given sequence classified as an enhancer by epigenomic marks is most likely to be involved in the regulation of the nearest active gene. Given the ability of enhancers to act over very large distances, however, this assumption seems dubious. In addition, vanishingly few enhancer sequences are ever subjected to the sort of genetic investigation to which we have subjected HS-3′β2 in this study, and so the proportion of “enhancers” identified by epigenomic marks or even functional assays that are actually required for normal gene regulation is unclear, as is the proportion of enhancers that are redundant. This question therefore represents a major future challenge in the study of distal transcriptional enhancers.

## Methods

### Ethics statement

This study was carried out in strict accordance with the recommendations in the Guide for the Care and Use of Laboratory Animals of the National Institutes of Health. The protocol was approved by the University Committee on Animal Resources of the University of Rochester Medical Center (Permit Number: 100393) at Rochester, New York.

### Cell Culture

Murine erythroid leukemia (MEL) cells [25] were grown at 37°C with a 5% CO_2_-humidified atmosphere. They were cultured in Dulbecco modified Eagle medium (DMEM, *Gibco*) containing 10% fetal bovine serum (*Gemini*), 1% PenStrep (*Gibco*) and 1% Glutamine (*Invitrogen*).

### Transient Reporter Gene Assay

The vector pGL3-Basic was used as the backbone for the firefly luciferase construct. The β1 or β2-globin gene promoters were cloned upstream of the luciferase gene. These sequences corresponded to coordinates +28393-29053 and +43271-44262, respectively, where +1 is set as the transcription start site (the first A in the sequence “GTACGTACTTGCTTCTG”) of the εy-globin gene. The HS-3′β1 (+32086-32259) or HS-3′β2 (+48788-48954) enhancers were cloned downstream of the luciferase gene in constructs containing the corresponding gene promoters. All promoters and enhancers were derived from the D allele of the murine β-globin locus (native to the 129/Svj and BALB/c strains). Murine erythroleukemia (MEL) 745a cells were co-transfected with a firefly luciferase construct and the control pRL-TK plasmid, which contains the renilla luciferase gene and is used to normalize each experiment for transfection efficiency. Transfections were performed using Lipofectamine LTX (*Invitrogen*) according to the manufacturer′s protocol. Luciferase expression was measured by luminescence at 48 hours post-transfection with the *Lumat* LB 9507 luminometer (Berthold Technologies USA), using the *Promega* Dual-luciferase Reporter Assay according to manufacturer instructions.

### Targeted deletions within the β-globin locus

Homologous recombination in murine embryonic stem cells and generation of mice from these cells was performed as previously described [6]. The selectable marker was removed by mating these mice with CMV-Cre transgenic mice [26]. The targeting vector for deletion of the β2-globin gene promoter consisted of a 0.81 kb fragment located 5′ of the β2-globin gene as the 5′ homology arm, a *loxP* site-flanked selectable marker (phosphoglycerate kinase I gene promoter driving the *neomycin* resistance gene), and a 4.10 kb fragment containing the 3′ portion of the β2-globin gene and additional downstream sequences as the 3′ homology arm. This results in the replacement of native sequences corresponding to +43653-44287, including the entire β2-globin gene promoter up to and including a small portion of coding sequence in the first exon, with the selectable marker cassette. The targeting vector for deletion of HS-3′β2 consisted of a 4.38 kb fragment (including part of the β2-globin gene) as the 5′ homology arm, the same *neomycin* marker cassette, and a 1.4 kb fragment of sequences located downstream of the β2-globin gene as the 3′ homology arm. This results in the replacement of native sequences corresponding to +48653-48968 (the location to which we have mapped HS-3′β2, and encompassing the entire sequence used in the transient reporter gene assay) with the marker cassette. The backbone for both targeting vectors consisted of pGEM-DTA, which contains the negative selection marker, diphtheria toxin A (DT-A), in a pGEM-3 backbone [20]. Sequences and maps that precisely delineate the homology arms, breakpoints for marker insertion and vector sequences are freely available upon request.

### Genotyping

Mouse tissue (tail) was digested overnight at 55°C with *Viagen* directPCR containing 0.5 mg/mL of proteinase K (*Promega*). The following morning the samples were incubated for 50 minutes at 85°C. Undigested material was removed by centrifugation for 1 minute at 13,000 rpm and the supernatant was removed and used for PCR. Genotyping PCR was performed using the HotStarTaq kit (*Qiagen*) with primers that either spanned the deletion or were located within it, in order to distinguish wildtype from mutants. In addition, primers that amplified the neomycin cassette were used to confirm Cre-mediated excision.

### Tissue collection

Mice were mated overnight and vaginal plugs were verified the next day to establish pregnancy; this was assumed to represent E0.5. At the depicted time points: E12.5, E14.5 or E16.5, pregnant mice were sacrificed by cervical dislocation for collection of embryonic peripheral blood and/or fetal liver. Individual embryos/fetuses were dissected and used for genotyping, mRNA expression analysis and ChIP.

### Expression analysis

RNA was isolated using the *Qiagen* RNeasy mini kit or Trizol (*Invitrogen*) following manufacturer instructions. cDNA was synthesized using iScript cDNA synthesis kit (*Biorad*). As a negative control, reverse transcription was also performed without enzyme. Quantitative real-time PCR (qPCR) was performed using iQSYBR Green Supermix (*Biorad*) according to manufacturer instructions, with the MyiQ Single Color Real Time PCR detection system and iCycler (*Biorad*). Primers were used to amplify εy-, βh1-, β1-, β2- and alpha1/2-globin cDNAs. We used 18 s mRNA as an internal control, and relative quantitation was performed by the 2(-Delta Delta C(T)) method. Primer sequences have been previously published and are available upon request. All measurements were normalized as the percentage of 18 s for plotting in graphs.

### Chromatin Immunoprecipitation (ChIP)

ChIPs were performed as previously described [6]. Protein G dynabeads (*Invitrogen*) were used to form a complex with the desired antibody. Antibodies used included: (1) normal rabbit IgG (*Millipore*), as a negative control, (2) anti-histone H3 acetyl Lys27 (*Active Motif*) and (3) anti-dimethyl histone H3 Lys4 (*Millipore*). Enrichments were calculated by using ΔCt to determine the ratio between amplification from IP and input DNA for a given test sequence, then dividing this by the average of the same ratio for the inactive controls. Inactive controls consisted of four non-erythroid gene promoters.

### Statistical analysis

Error bars represent standard error of the mean. When appropriate, student t-test was used to determine significant difference (*p* value) between the mean of the number of data points (n) shown.
